# Cross-Frequency Coupling Between Cerebral Blood Flow Velocity and EEG in Ischemic Stroke Patients With Large Vessel Occlusion

**DOI:** 10.3389/fneur.2019.00194

**Published:** 2019-03-12

**Authors:** Xiuyun Liu, Yuehua Pu, Dan Wu, Zhe Zhang, Xiao Hu, Liping Liu

**Affiliations:** ^1^Department of Physiological Nursing, University of California, San Francisco, San Francisco, CA, United States; ^2^Neurointensive Care Unit, Department of Neurology, Beijing Tiantan Hospital, Capital Medical University, Beijing, China; ^3^China National Clinical Research Center for Neurological Diseases, Beijing Tiantan Hospital, Capital Medical University, Beijing, China; ^4^School of Computer and Information Technology, Beijing Jiaotong University, Beijing, China; ^5^Department of Neurosurgery, School of Medicine, University of California, Los Angeles, Los Angeles, CA, United States; ^6^Department of Neurological Surgery, University of California, San Francisco, San Francisco, CA, United States; ^7^Institute of Computational Health Sciences, University of California, San Francisco, San Francisco, CA, United States

**Keywords:** cerebral blood flow, EEG, cross frequency coupling, stroke, neurovascular coupling

## Abstract

**Background:** Neurovascular coupling enables a rapid adaptation of cerebral blood flow (CBF) to support neuronal activities. Whether this mechanism is compromised during the acute phase after ischemic stroke remains unknown. In this study, we applied a phase-amplitude cross-frequency coupling (PAC) algorithm to investigate multimodal neuro signals including CBF velocity (CBFV), and electroencephalography (EEG).

**Methods:** Acute ischemic stroke patients admitted to the Neurointensive Care Unit, Tiantan Hospital, Capital Medical University (Beijing, China) with continuous monitoring of 8-lead EEG (F3-C3, T3-P3, P3-O1, F4-C4, T4-P4, P4-O2), non-invasive arterial blood pressure (ABP), and bilateral CBFV of the middle cerebral arteries or posterior cerebral arteries were retrospectively analyzed. PAC was calculated between the phase of CBFV in frequency bands (0–0.05 and 0.05–0.15 Hz) and the EEG amplitude in five bands (δ, θ, α, β, γ). The global PAC was calculated as the sum of all PACs across the six EEG channels and five EEG bands for each patient. The hemispherical asymmetry of cross-frequency coupling (CFC) was calculated as the difference between left and right PAC.

**Results:** Sixteen patients (3 males) met our inclusion criteria. Their age was 60.9 ± 7.9 years old. The mean ABP, mean left CBFV, and mean right CBFV were 90.2 ± 31.2 mmHg, 57.3 ± 20.6 cm/s, and 68.4 ± 20.9 cm/s, respectively. The PAC between CBFV and EEG was significantly higher in β and γ bands than in the other three bands. Occipital region (P3-O1 and P4-O2 channels) showed stronger PAC than the other regions. The deceased group tended to have smaller global PAC than the survival group (the area under the receiver operating characteristic curve [AUROC] was 0.81, *p* = 0.57). The unfavorable outcome group showed smaller global PAC than the favorable group (AUROC = 0.65, *p* = 0.23). The PAC asymmetry between the two brain hemispheres correlates with the degree of stenosis in stroke patients (*p* = 0.01).

**Conclusion:** We showed that CBFV interacts with EEG in β and γ bands through a phase-amplitude CFC relationship, with the strongest PAC found in the occipital region and that the degree of hemispherical asymmetry of CFC correlates with the degree of stenosis.

## Introduction

The brain is only able to withstand transient blood supply disruption. Adequate cerebral blood flow (CBF) must be maintained to ensure a constant delivery of oxygen and substrates and to remove the waste products of metabolism (1). This control system involves neurogenic, metabolic, myogenic, and endothelial mechanisms, but is still poorly understood (2, 3). Neurovascular coupling is an effective intrinsic vasoregulative mechanism that rapidly adapts CBF in accordance with neuronal activity (4). Its dysfunction has been implicated in serious neurological conditions, such as ventricular hemorrhage, ischemic stroke, hypertension, Alzheimer disease, subarachnoid hemorrhage, etc. (3, 5–7). With modern techniques in neuro critical care units (NCCU), monitoring of neuronal activity through electroencephalography (EEG) and cerebral hemodynamics through transcranial Doppler (TCD) has become available. In the late 1970s, Sharbrough et al. found a strong correlation between CBF and alterations in EEG during carotid occlusion (8); and Vespa et al. proved that the relative alpha variability of EEG was reduced with cerebral vasospasm in SAH patients (9); Additionally, Foreman and Claassen summarized that when normal CBF declines, the EEG first loses its faster frequencies, then activity with slower frequencies gradually increases, and if CBF continues to decline, the EEG ultimately falls silent, and cellular damage becomes irreversible (8, 10–12). Nevertheless, the precise mechanism of how neuronal activation interacts with CBF is still not fully understood. In particular, there is limited data of neurovascular coupling from stroke patients.

In the field of electroneurophysiology, there has been particular interest in how low frequency brain signal oscillations modulate high frequency oscillations, because recent evidence suggests a functional role for this type of cross-frequency coupling (CFC) (13, 14). The low frequency oscillations are associated with modulating activity in long range communication and long temporal windows (global process), while the high frequency rhythms modulate activity in small regions and short time windows (local process) (15, 16). This interaction of rhythms in different frequency bands mainly includes three types: amplitude-to-amplitude coupling (AAC), phase-to-phase coupling (PPC), and phase-to-amplitude coupling (PAC) (16–19). PAC, in which the phase of a low frequency rhythm from one signal regulates the amplitude of higher frequency activity (either from the same or another signal), is identified as the main communication mechanism of EEG in frequencies below 80 Hz (20).

**Figure 1:**
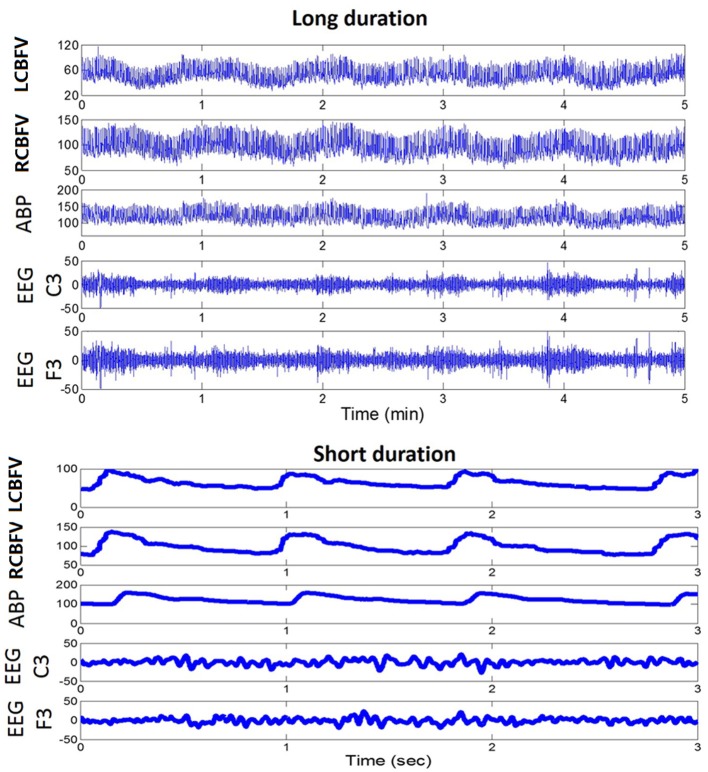
An example of a daily recording of CBFV, ABP, and EEG in a neuro critical care unit. Upper panel: recordings of fast fluctuations (5 min). Lower panel: recordings of signal waveforms. CBFV, cerebral blood flow velocity; LCBFV, left CBFV; RCBFV, right CBFV; ABP, arterial blood pressure; EEG, electroencephalography.

In our daily recording of EEG and CBF velocity (CBFV), we have visually noticed an interesting relationship between the phase of slow waves of CBFV and the amplitude of high frequency EEG components, as shown in Figure 1 (long duration). Therefore, we sought to find out whether PAC relationship exists between EEG and CBFV and whether the neurovascular coupling characteristics can be described using these two signals. In particular, we have three hypotheses: (1) there is a relationship between EEG and CBFV following ischaemic stoke, which might inform on the status of neurovascular coupling; (2) the degree of asymmetry of neurovascular coupling of the two brain hemispheres is positively related with the degree of occlusion on the patients with unilateral occlusion in major arteries; (3) neurovascular coupling is weaker in the deceased group than in the survival group.

## Methods and Materials

A total of twenty recordings from seventeen stroke patients admitted to Neurointensive Care Unit, Department of Neurology, Beijing Tiantan Hospital, Capital Medical University (Beijing, China) were studied. Three patients were monitored twice on the 2nd day of admission and the 4th day of admission. The other patients were only monitored once. The inclusion criteria were as follows: (1) men or women aged ≥18 years; (2) acute ischemic stroke confirmed by computed tomography (CT), or magnetic resonance imaging (MRI) of the head; (3) stroke onset within 24 h of hospital admission; (4) stroke caused by cerebral large artery occlusion, including internal carotid artery (ICA), middle cerebral artery (MCA), and vertebral or basilar artery (BA), and (5) patient received recanalization therapy. We excluded the patients who had an insufficient or absent acoustic temporal bone window and who had scalp wounds or infections, as EEG was not tolerated in those cases. All the enrolled patients underwent continuous monitoring of 8-lead EEG, non-invasive arterial blood pressure (ABP), and CBFV of the bilateral MCA or posterior cerebral arteries (PCA). Written consent form was obtained from each patient or the next of kin. The study was approved by local Institutional Review Board (IRB). Patients' NIH Stroke Score (NIHSS) were recorded on admission and at discharge. Patients' Modified Rankin Scale (mRS) for neurologic disability was recorded at discharge. One stroke patient recording was excluded due to poor data quality.

We monitored bilateral CBFVs from the MCA for the anterior circulation stroke patients (*n* = 13) and CBFVs of PCA (*n* = 3) for posterior circulation stroke patients through 2-MHz probes mounted on a headband using transcranial Doppler (Doppler BOX, DWL, Singen, Germany or EMS-9PB Transcranial Doppler Ultrasound System, Delica, China). ABP was continuously monitored through non-invasive finger plethysmography (CNAP Monitor 500, Graz, Austria or Finometer model 1, Finapres, Netherlands). Continuous EEG monitoring was performed using an 8 electrode longitudinal bipolar montage (Nicolet V44 EEG Monitor, Natus Neurology Incorporated, Wisconsin, USA or NSD-7101 Neuro Monitor System, Delica, China) with electrodes placed according to the international 10–20 system (F3-C3, T3-P3, P3-O1, F4-C4, T4-P4, P4-O2) (21). The CBFV, ABP, and EEG signals were saved simultaneously through the neuro monitor system at a sampling frequency of 500 Hz.

### Estimation of Phase-Amplitude Cross-Frequency Coupling (PAC)

PAC was calculated between the phase of slow waves of CBFV (0–0.05 and 0.05–0.15 Hz) and the amplitude of five EEG bands (δ, θ, α, β, γ). We used the method proposed by Canolty et al. (17) to extract PAC through a complex valued signal: ${\text{A}}_{\text{EEG}}\left(t\right){\text{e}}^{\text{i}{\text{φ}}_{\text{CBFV}}\left(t\right)}$, where A_EEG_(*t*) refers to amplitude of EEG in each frequency band, and φ_CBFV_(*t*) refers to the phase of CBFV. In order to calculate PAC, the raw EEG signal was first separated into bands with center frequencies ranging from 2 to 44 Hz, in 2 Hz steps, with 2 Hz bandwidths. This process created a set of real valued band-pass filtered EEG signals {*EEG*(*t*)}. Second, the Hilbert Transform was applied to each signal in {φ_CBFV_(*t*)} to create a set of complex-valued analytic signals {*Z*_EEG_(*t*)}. The absolute value of each analytic signal was then obtained to produce the set of analytic amplitude time series {A_EEG_(*t*)}. Next, the CBFV signals were separated into two bands: 0–0.05 and 0.05–0.15 Hz, and the phase information of CBFV in these two frequencies was extracted from the Hilbert Transform, resulting in {φ_CBFV_(*t*)}. We then constructed a composite complex-valued signal by combining the amplitude of EEG of one frequency band and the phase series of CBFV of another frequency band: ${z\left(t\right)=A}_{\text{EEG}}\left(t\right){\text{e}}^{\text{i}{\text{φ}}_{\text{CBFV}}\left(t\right)}$. This composite signal takes on some particular value in the complex plane at each time point. If the probability density function (PDF) of *z*(*t*) is not radially symmetric, then it must be the case that either (1) A_EEG_ and φ_CBFV_ share mutual information, or (2) the distribution of φ_CBFV_ is non-uniform. Measuring the degree of asymmetry of this PDF, which can be done by computing the mean or first moment M of *z*(*t*), provides a useful metric of coupling between the two time series (14, 17).

Since the question of interest is the degree of coupling between A_EEG_ and φ_CBFV_, rather than the statistical properties of either A_EEG_ or φ_CBFV_ examined alone, the mean M must first be normalized before it can be used as a metric of coupling strength (17). In other words, we were interested in the properties of the joint distribution of A_EEG_ or φ_CBFV_. One way to accomplish this is to compare the actual mean M (*M*_*real*_) to a set of surrogate means {*M*_*sur*_} created by offsetting A_EEG_ or φ_CBFV_ by some large time lag. That is, we can introduce a time lag τ between A_EEG_ and φ_CBFV_ such that the composite signal is a function of both time and lag: ${z\left(t,\;\tau \right)=A}_{\text{EEG}}\left(t+\tau \right){\text{e}}^{\text{i}{\text{φ}}_{\text{CBFV}}\left(t\right)}$. Note that the dependence (if any) between A_EEG_ and φ_CBFV_ will be a function of the lag τ between them, decreasing for large τ, while τ has no effect on the distribution of φ_CBFV_ alone or A_EEG_ alone. Therefore, computing the distribution of *z*(*t*, τ) at large τ can maintain the statistics of the individual time series, and only the pairing of sample points between the two time series is changed. Any asymmetry in the distribution of *z*(*t*, τ) at large τ will be due to the non-uniformity of φ_CBFV_, while the scale (how far points fall from the origin) will be determined by A_EEG_ alone. The modulus or length of *M*_*real*_, compared to the distribution of surrogate lengths, provides a measure of the coupling strength, while the angle of M, compared to the distribution of surrogate angles, indicates the phase of CBFV associated with the largest EEG amplitudes (14). We can define a normalized or z-scored length M_norm_ = (M_real_ − μ)/σ, where μ is the mean of the surrogate lengths and σ is their standard deviation. This normalization ensures that *M*_*norm*_ is insensitive to the marginal distributions of A_EEG_ and φ_CBFV_ and is sensitive only to their joint distribution, as desired. We define this normalized metric *M*_*norm*_ as the modulation index (MI) for PAC assessment in this paper. It is thus assumed that the existence of coupling leads to a larger MI (14).

After calculating the MIs between the phase of CBFV in two bands (0–0.05 and 0.05–0.15 Hz) and the amplitude of all the EEG bands (2 to 44 Hz, in 2 Hz steps, with 2 Hz bandwidths), the MIs were averaged in five EEG frequency bands: δ (1–4 Hz), θ (4–7Hz), α (7–13 Hz), β (13–30 Hz), and γ (30–45 Hz). Therefore, for each EEG channel, we obtained 5 × 2 = 10 MI values for CBFV of each side at every calculating time point, where 5 refers to the five EEG frequency bands (δ, θ, α, β, γ) and 2 refers to the two CBFV frequency bands. The MI was calculated using a 300 s window, updated every 2 min.

### Global PAC

In order to assess the systematic coupling between CBFV and EEG, we introduced a parameter named global PAC. First, the MIs between each EEG channel (6 channels) and CBFV of each side were averaged across the whole recording time, which resulted in 6 × 2 × 10 = 120 MI values for each patient (2 refers to the CBFV of two sides, 6 refers to six EEG channels and 10 refers to the MI values that each EEG channel can produce). The global PAC was calculated as the sum of all the 120 MI values.

### Degree of Asymmetry of Bilateral PACs

In order to evaluate the degree of asymmetry of bilateral PACs, we first calculated the mean value of MIs between left CBFV (*MI*_*left*_) and the 6 EEG channels (*n* = 6 × 10 = 60), and the mean value of MIs between right CBFV (*MI*_*right*_) and all the 6 EEG channels (*n* = 6 × 10 = 60). The absolute difference between *MI*_*left*_ and *MI*_*right*_ was calculated to demonstrate the degree of asymmetry of bilateral PACs. We hypothesized that the degree of asymmetry of bilateral PACs is positively related with the degree of stenosis.

### Collateral Flow Strength

We calculated the mean value of MI (*MI*_*ips*_), between CBFV on the side contralateral to stroke and the EEG of the ipsilateral side (3 channels), and the mean value of MI (*MI*_*con*_) between CBFV on the side contralateral to stroke and the EEG on the side of stroke (3 channels). The difference between *MI*_*ips*_ and *MI*_*con*_ was calculated to denote the collateral flow strength. We hypothesized that smaller difference between these two metrics might correspond to more efficient collateral flow. The difference was compared with the degree of stenosis.

### Statistical Analysis

The statistical analyses were performed using Matlab software (ver. R2012A, MathWorks, Inc.). In order to determine which frequency band showed the strongest coupling relationship between EEG and CBFV, the mean MI across all the EEG channels (six channels) was calculated for each EEG frequency band (δ, θ, α, β, γ) individually, resulting in five MI values in each CBFV band at each side. Then in order to eliminate individual differences and make the comparison compatible, each MI was divided by the sum of the five MIs for each patient. Finally, all the patients' normalized MIs were separated into five groups with each group representing one EEG band. A One Way ANOVA was used to tell whether there is significant difference between at least two bands. If the *p* value of One Way ANOVA was smaller than 0.05, an additional multiple comparison test (Bonferronim) was used to find out where the significant difference was located.

In order to identify the region of the brain that showed the strongest PAC between CBFV and EEG, the average value of MIs across all EEG bands was calculated for each EEG channel, resulting in six MI values for each patient in each CBFV band at each side. Then each MI value was divided by the sum of the six MIs of each patient for normalization. Finally, all the patients' normalized MIs were separated into 6 groups with each group representing one EEG channel. A One Way ANOVA was used to tell whether there is significant difference between at least two channels. If the *p* value of One Way ANOVA was smaller than 0.05, an additional multiple comparison test (Bonferronim) was used to find out where the significant difference was located.

Patients were divided into a deceased (mRS = 6) and a survival group (mRS = 1 to 5), and into a favorable (mRS: 1 to 2) and an unfavorable group (mRS: 3 ~ 6) (22). The mean global PAC in each group was calculated. The Wilcoxon rank sum test was used to calculate the difference in the global PAC between the deceased and survival groups, and between the favorable and unfavorable groups. *p* < 0.05 was considered to be significant. Receiver Operating Characteristic (ROC) curves were used to compare the ability of PAC in distinguishing patient outcome, rendering an area under the ROC curve (AUROC). The patients were also divided into three groups according to NIHSS at discharge: NIHSS < 10, 10 ≤ NIHSS < 20 and 20 ≤ NIHSS. Mean global PAC was calculated in each group and a One Way ANOA was used to compare the difference among the three groups.

## Results

### Patient Demographics

The mean age of the 16 (three males) patients enrolled in the study was 60.9 ± 7.9 (mean ± SD) years. Nineteen recordings were studied, with a mean duration of 100.0 ± 42.6 min, ranging from 15.3 to 172.9 min. Information about each patient's blocked artery, age, NIHSS on admission and at discharge, mRS at discharge, mean ABP, and mean CBFV of both sides are summarized in Table 1.

**Table 1:** Patients' demographic.

**Patient**	**Sex**	**Blocked Artery**	**Age**	**NIHSS on admission**	**NIHSS at discharge**	**mRS at discharge**	**ABP (mmHg)**	**Left CBFV (cm/s)**	**Right CBFV (cm/s)**
1	M	RICA	57	11	10	4	113.0 ± 32.1	55.7 ± 13.8	91.3 ± 18.0
2	F	LICA	85	20	35	6	52.3 ± 13.1	87.8 ± 35.8	44.9 ± 20.7
3	M	RICA	54	40	34	5	85.9 ± 26.4	64.4 ± 14.8	118.9 ± 30.4
4	M	BA	46	21	10	5	105.5 ± 24.8	54.3 ± 10.0	63.3 ± 17.5
5	M	LICA	75	26	19	5	111 ± 12.0	50.0 ± 20.6	99.2 ± 50.1
6	F	LICA	56	14	4	4	90.3 ± 16.7	22.8 ± 13.3	49.5 ± 15.4
7	M	LICA	62	18	11	4	120.4 ± 23.4	38.4 ± 9.5	61.4 ± 14.7
8	M	LICA	68	15	11	4	99.6 ± 23.9	75.2 ± 33.7	68.6 ± 19.2
9	M	RICA	58	13	9	4	104.2 ± 22.9	95.6 ± 36.4	61.1 ± 21.4
10	F	BA	64	37	35	5	98.0 ± 25.9	77.1 ± 19.0	73.9 ± 18.6
11	M	LMCA	66	15	1	1	99.3 ± 27.5	57.6 ± 18.5	38.0 ± 13.8
12	M	RICA	76	10	35	6	114.4 ± 41.1	23.5 ± 22.5	64.5 ± 18.6
13	M	LMCA	62	18	34	5	98.9 ± 29.1	51.2 ± 13.0	57.6 ± 17.7
14	M	RICA	56	13	2	1	97.2 ± 13.9	40.1 ± 10.0	69.7 ± 30.3
15	F	RMCA	57	15	16	5	115.8 ± 21.5	56.5 ± 17.4	52.2 ± 16.2
16	M	BA	45	NA	NA	NA	47.8 ± 11.7	67.4 ± 16.7	80.6 ± 20.7

*NIHSS, NIH stroke scale; mRS, Modified Rankin Scale for neurologic disability; NA, not available; ICA, internal carotid artery; MCA, middle cerebral artery; BA, basilar artery; L, left side; R, rite side. ABP, arterial blood pressure; CBFV, cerebral blood flow*.

### How CBFV and EEG Interact With Each Other

**Figure 2:**
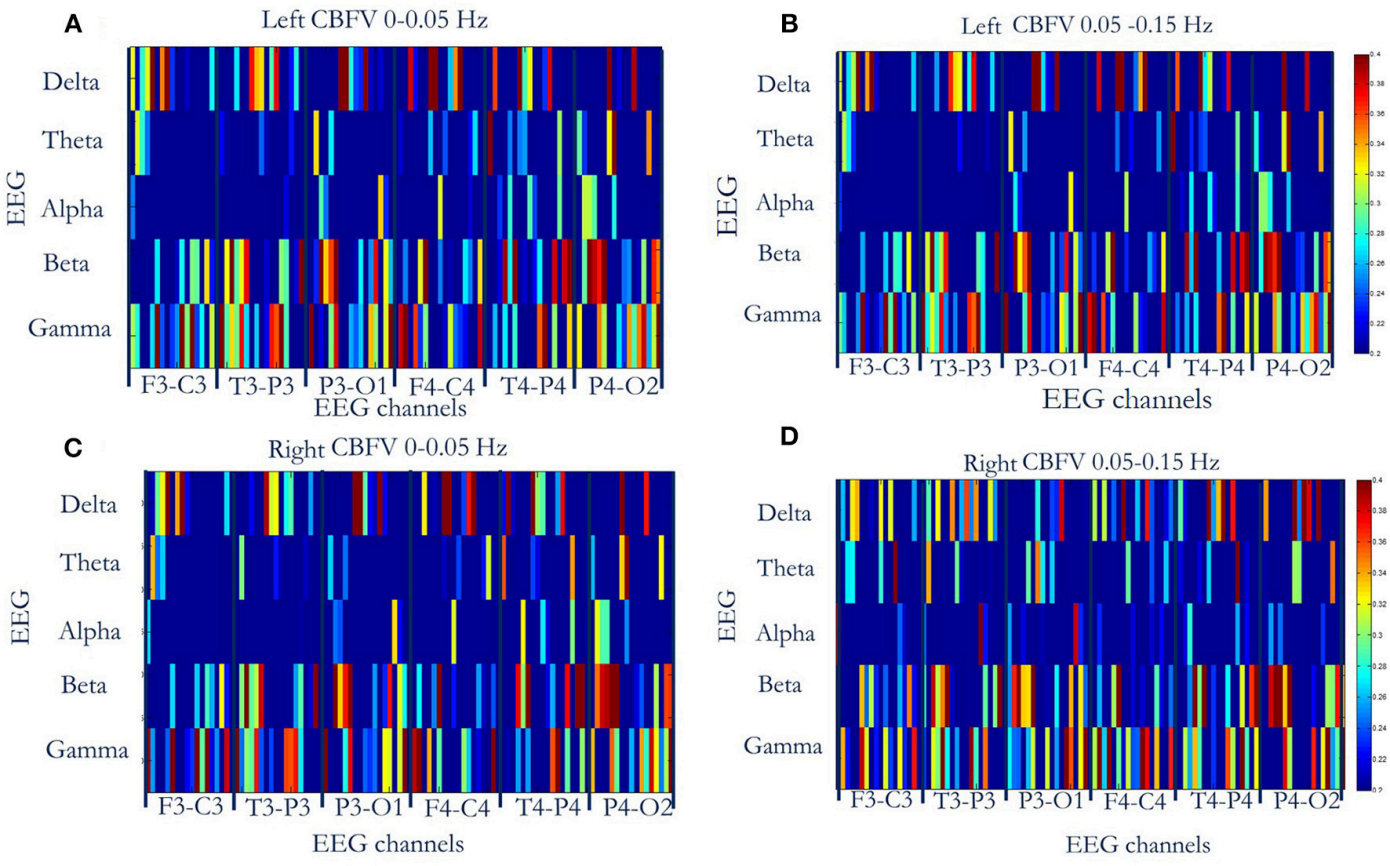
Phase-amplitude cross-frequency coupling (PAC) between CBFV and EEG of six channels (F3-C3, T3-P3, P3-O3, F4-C4, T4-P4, P4-O2) of all the 16 patients. The *x* axis represents six EEG channels, and the *y* axis represents five EEG bands. Each subfigure represents CBFV of one side in one frequency (**A**,**C**: 0~0.05 Hz and **B,D**:0.05~0.15 Hz), and one dot represents one patient, with red color indicating strong PAC and blue color indicating weak PAC. In general, PAC between CBFV and EEG in β and γ bands show brighter belt, indicating stronger PAC in these two bands than the other three EEG bands. **(A)** PAC between phase of left CBFV (0–0.05 Hz) and amplitude of EEG (six channels, five bands). **(B)** PAC between phase of left CBFV (0.05–0.15 Hz) and amplitude of EEG. **(C)** PAC between phase of right CBFV (0–0.05 Hz) and amplitude of EEG. **(D)** PAC between phase of right CBFV (0.05–0.15 Hz) and amplitude of EEG. CBFV, cerebral blood flow velocity; ABP, arterial blood pressure; EEG, electroencephalography.

**Figure 3:**
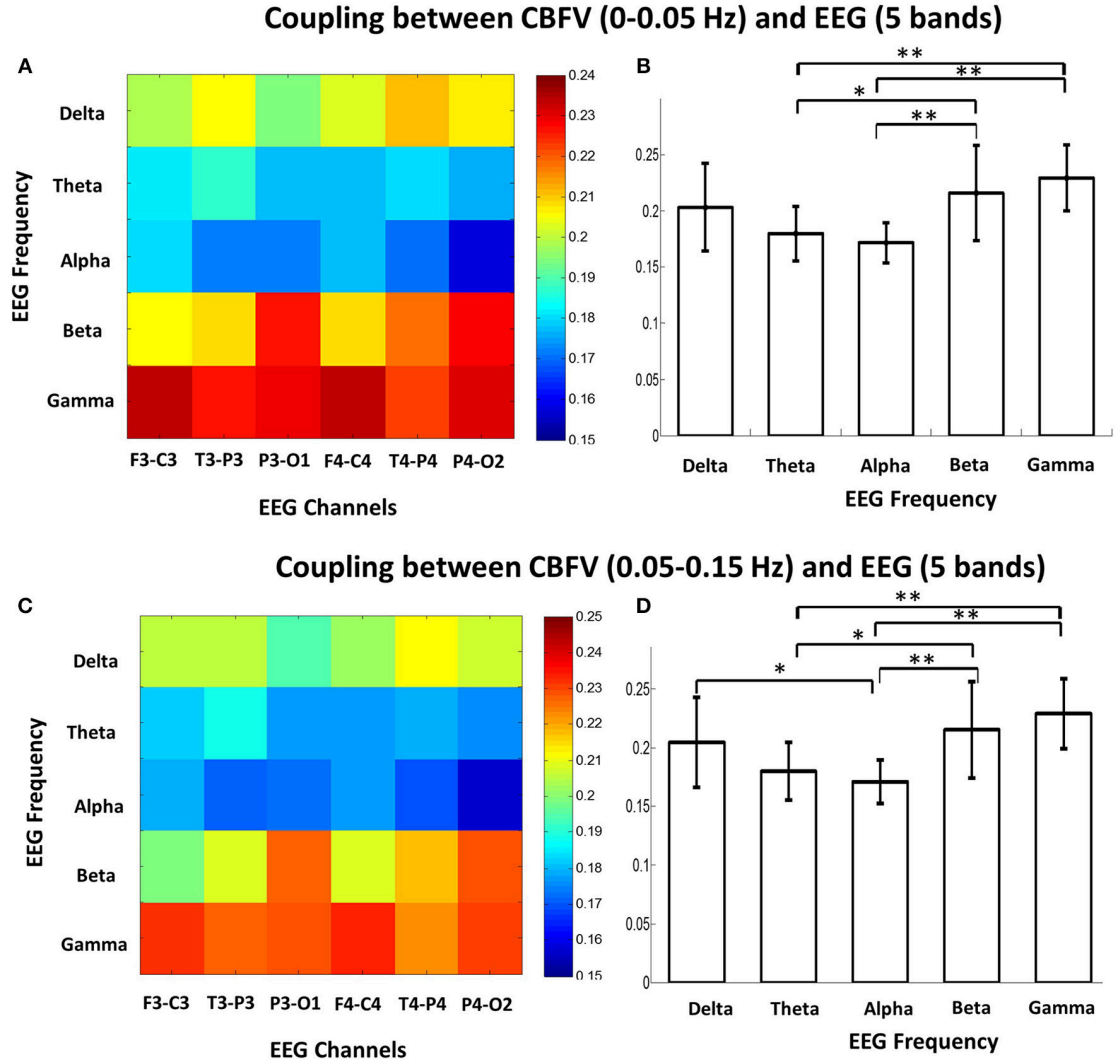
**(A)** Mean phase-amplitude cross-frequency coupling (PAC) between CBFV (0–0.05 Hz) and EEG of six channels in five frequency bands (δ, θ, α, β, γ) of the 16 patients. **(B)** Statistical comparison of mean PAC between CBFV (0–0.05 Hz) and EEG in the 5 frequency bands (δ, θ, α, β, γ). The PAC in β and γ bands were significant higher than the other bands. One star means *p* < 0.05; two stars means *p* < 0.01. **(C)** Mean PAC between CBFV (0.05–0.15 Hz) and EEG of six channels in five frequency bands of the 16 patients. **(D)** Statistical comparison of mean PAC between CBFV (0.05–0.15 Hz) and EEG in the 5 frequency bands. The PAC in β and γ bands were significant higher than the other bands. CBFV, cerebral blood flow velocity; ABP, arterial blood pressure; EEG, electroencephalography.

The MIs between the phase of CBFV (0–0.05 Hz and 0.05–0.15 Hz) and EEG amplitude in each band (five bands: δ, θ, α, β, γ) were calculated and averaged across the whole recording period for each EEG channel. Figure 2 shows the MIs of all 16 patients, where the *x* axis represents six EEG channels, and the *y* axis represents five EEG bands. Each subfigure represents CBFV of one side in one frequency (A, C: 0~0.05 and B, D:0.05~0.15 Hz), and one dot represents one patient, with red color indicating strong PAC and blue color indicating weak PAC. In general, MIs between CBFV and EEG in β and γ bands show brighter belt, indicating stronger PAC in these two bands than the other three EEG bands (Figure 2). The One Way ANOVA and multiple comparison test of normalized MIs show that PAC is significantly stronger in EEG β and γ bands than in the other three bands (Figure 3).

### The Brain Region That Shows Strongest PAC Between CBFV and EEG

**Figure 4:**
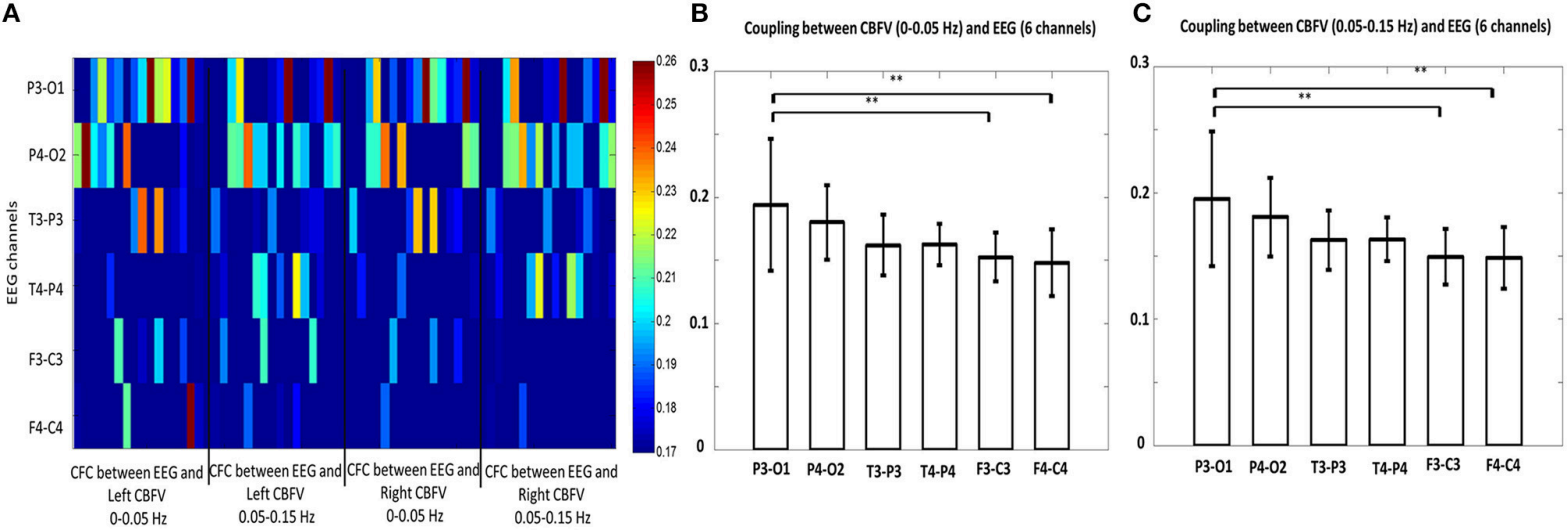
**(A)** Phase-amplitude cross-frequency coupling (PAC) between bilateral CBFVs and EEG of six channels (F3-C3, T3-P3, P3-O3, F4-C4, T4-P4, P4-O2). The x axis represents the bilateral CBFV frequency bands (0–0.05 and 0.05–0.15 Hz), and the y axis represents six EEG channels (F3-C3, T3-P3, P3-O1, F4-C4, T4-P4, P4-O2). One point represents one patient, with red color indicating strong PAC and blue color indicating weak PAC. In general, P3-O1 and P4-O2 show brighter belts than the other four EEG channels. **(B)** Statistical comparison of mean PAC between CBFV (0–0.05 Hz) and EEG of 6 channels. **(C)** Statistical comparison of mean PAC between CBFV (0.05–0.15 Hz) and EEG of 6 channels. The PAC of P3-O1 channel was significant higher than the other channels. CBFV, cerebral blood flow velocity; ABP, arterial blood pressure; EEG, electroencephalography. Two stars indicates *p* < 0.05.

We also investigated the brain region that shows strongest phase-amplitude coupling between CBFV and EEG. Figure 4A combines the MIs of all the 16 patients together, where the x axis represents the bilateral CBFV frequency bands (0–0.05 and 0.05–0.15 Hz), and the y axis represents six EEG channels (F3-C3, T3-P3, P3-O1, F4-C4, T4-P4, P4-O2). One point represents one patient. In general, P3-O1 and P4-O2 show brighter belts than the other four EEG channels (Figure 4A). The One Way ANOVA and multiple comparison test indicate stronger PAC in the occipital region (P3-01, P4-02) than the other regions (Figures 4B,C), with MI in P3-O1 channel significantly bigger than other channels (Figures 4B,C, *p* < 0.05). The relationship between PAC and outcome.

**Figure 5:**
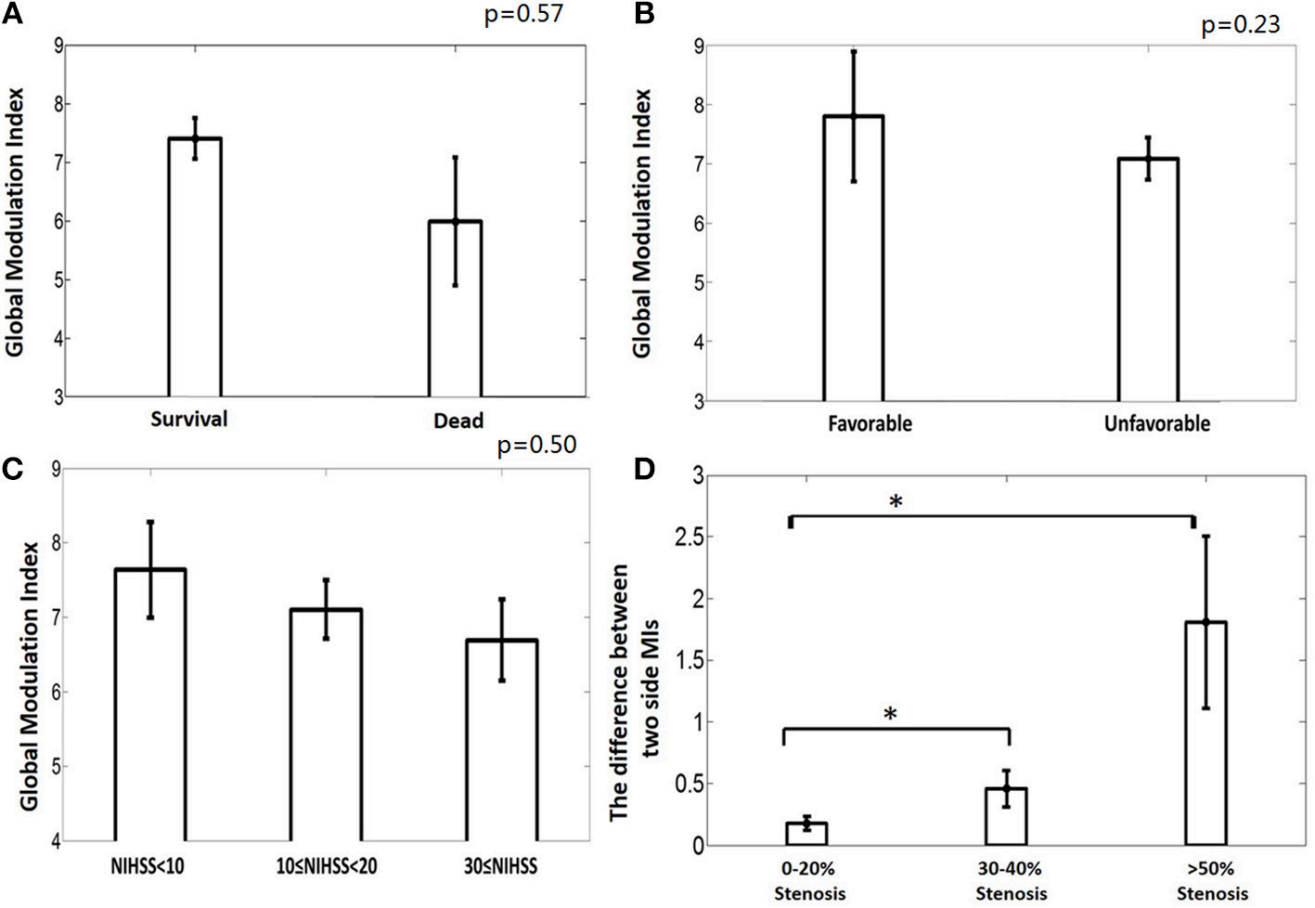
**(A)** The deceased group tends to have weaker phase-amplitude coupling (PAC) relationship between CBFV and EEG than the survival group, though not significantly. **(B)** The unfavorable group tends to have weaker PAC than the favorable group, though not significantly. **(C)** The group with higher NIHSS has weaker global PAC; **(D)** Greater degree of occlusion in unilateral main artery, demonstrated by higher degree of stenosis, is associated with greater asymmetry in the coupling relationship between the two brain hemispheres. MI: modulation index, which is used to indicate the strength of PAC. One star means *p* < 0.05.

The patients were divided into a deceased (*n* = 2) and a survival (*n* = 14) group. The mean MI of the deceased group was 5.99 ±1.54 and the mean MI of the survival group was 7.36±1.19. No significant difference exists between these two groups (AUROC = 0.81, *p* = 0.57, Figure 5A). There is no significant difference between the mean MI of the favorable group (*n* = 2, mean MI was 7.8 ±1.55) and unfavorable group (*n* = 14, mean MI was 7.1 ± 1.27, AUROC = 0.65, *p* = 0.23, Figure 5B), either. The global PAC seemed to increase while NIHSS increased, but this relationship was not significant (*p* = 0.50, Figure 5C).

The relationship between the degree of asymmetry of the bilateral PACs and the degree of occlusion is interesting (Figure 5D). Greater degree of occlusion in unilateral main artery, demonstrated by higher degree of stenosis, was associated with greater asymmetry in the coupling relationship between the two brain hemispheres (mean difference of bilateral PACs was 0.27 ± 0.24, 0.46 ± 0.36, and 1.81 ± 1.21 at 0–20% stenosis, 30–40% stenosis, and >50% stenosis, *p* = 0.01).

No significant relationship was found between the collateral flow strength and the degree of stenosis (mean *MI*_*ips*_ - *MI*_*con*_ was 0.07 ± 0.08, 0.08 ± 0.23, 0.33 ± 0.32 at 0–20, 30–40, and >50% stenosis, *p* = 0.19).

## Discussion

As the first leading cause of death in developing countries, stroke is the greatest cause of disability (23, 24). Strokes are classified as hemorrhagic and ischemic, with the majority falling into the latter category (25). Studies show that CBF decreases in the occluded artery which is the main etiology of ischemia (26). However, the effect of the occlusion in large intracranial arteries and the related hemodynamic changes are still not clear in stroke patients (27). The present work demonstrates that it is possible to link the degree of occlusion in unilateral main artery and the asymmetry of neurovascular coupling strength between the two brain hemispheres. In summary, we have shown strong coupling relationship between the phase of CBFV slow waves and the amplitude of EEG in β and γ bands. We also found the occipital region displayed the strongest PAC among the limited number of brain regions studied. In this cohort of stroke patients, the degree of stenosis was positively related with the asymmetry of the coupling relationship between the two brain hemispheres. Moreover, the deceased group and unfavorable outcome groups tended to have a weaker neurovascular coupling relationship than the survival or favorable group, though this was not significant.

A close correlation has been established between EEG and regional CBF in experimental animals and in humans under normal conditions (28–30). This relationship is reasonable because both EEG and CBF are closely related with neuronal metabolic states (31). Increased neurological activities, such as seizures and arousal, need more CBF and increased cerebral metabolism. It has been demonstrated that under normal conditions, local field potential activity, and in particular the γ-band component, is thought to be a more reliable predictor of perfusion based signals (32–34). However, how EEG and CBF interact with each other in stroke patients remains unknown. In this study, we found a close CFC relationship between EEG and CBFV in a cohort of ischemic stroke patients. In general, CBFV phase in low frequency interacts with EEG amplitude in high frequencies (β and γ bands). The present findings closely link hemodynamic responses to the process of EEG oscillations in β and γ bands. Our results are compatible with previous findings (32–35). Scheeringa et al. found γ band EEG power correlates positively with the superficial layers' hemodynamic signal and that β power is negatively correlated to deep layer hemodynamics (36). A possible explanation of the relationship between γ band EEG and CBFV is that γ oscillations, mainly observed in granular and supragranular layers, are thought to be initiated by the firing of inhibitory interneurons and pyramidal cells (34, 36). At the same time, these interneurons contain enzymes for the synthesis of vasoactive compounds such as nitric oxide (NO) and vasoactive peptides (34, 37, 38). Thus, when cortical networks engage in γ oscillations, inhibitory interneurons are highly active, and as their discharges are phase-locked to the oscillations (23), their activity increases with oscillation frequency. Moreover, previous findings also found that β band EEG is inversely correlated to hemodynamic signals and is predominantly measured in deep layers (i.e., the infragranular layers) (39–41). The deep layers are therefore also a likely source for the β signal and vascular mediators. Moreover, since the feedforward connections of the brain target the granular layer (42) and the feedback connections originate preferentially in the infragranular layers (43), we hypothesize that the synchronization between CBF and EEG γ band might reflect feedforward influences, and the synchronization between CBF and EEG β band might reflect feedback influences of CBF control (44). However, these hypotheses are highly speculative given that we are studying this phenomenon at a super macro level.

Our data also shows that the strongest phase-amplitude coupling between CBFV and EEG is found in the occipital region. The occipital lobe, the cerebellum, and the medial aspects of the temporal lobe receive blood from the vertebrobasilar system, which is called the posterior circulation (36). It has already been noted that in human EEG, γ rhythms are prevalent in local visual response synchronization (45, 46). In his article, Scheeringa pointed out that γ band modulation dominated in posterior electrodes consistent with a source in the early visual cortex. Furthermore, Bastos et al. found that among primate visual cortical areas, feedforward communication utilizes the θ and γ-bands and feedback communication relies upon the β band (44). These findings offer some possible explanations of why we observed stronger CFC in P3-O1 and P4-O2 channel in the present study.

Acute ischemic stroke is a leading cause of morbidity and mortality worldwide (47). It has been demonstrated that large vessel occlusion related acute ischemic strokes are associated with more severe deficits and have worse long-term outcomes (48). Stenosis or occlusion of the major arteries of the head and neck may cause hemodynamic impairment of the distal cerebral circulation (49). The key in reducing the high morbidity and mortality associated with stroke is to develop a method that can detect cerebral asymmetries and vessel occlusion. Several neuroimaging methods are currently available for the indirect assessment of the hemodynamic effect of atherosclerotic stenosis or occlusion on the distal cerebral vasculature. However, these methods are not continuous. It will be of great interest to develop a tool that can identify the degree of patient's stenosis continuously at bedside. Our study demonstrated the potential of using PAC between CBFV and EEG as an indicator of the degree of occlusive level in stroke patients. Higher stenosis is positively related with bigger difference between the bilateral PACs. For a patient with occlusion in the unilateral big artery, the blood supply will mainly rely on the contralateral, unblocked artery. If the artery cannot supply sufficient blood to both hemispheres, it will cause asymmetry of the coupling relationship between the two brain hemispheres. The more severely the artery is blocked, the more obvious this asymmetry is.

This study is a preliminary research to develop and validate metrics for neuro vascular coupling assessment using continuously available signals at the bedside. Our current focus on studying the relationship between EEG amplitude and CBFV phase was motivated by the observation as presented in Figure 1 where one can appreciate the changes of EEG amplitude are related to changes in phase of slow waves of CBFV. Physiologically speaking, EEG activity is much faster than hemodynamic changes. Therefore, the time delay (or equivalently the phase relationship) would be a critical aspect of neurovascular coupling. However, coherence may be also of interest as a potential marker of neuro vascular coupling. One potential challenge in using coherence is that it only provides the strength of relationship at matched frequencies of two signals, which might be a disadvantage because relevant information in EEG is at a higher frequency than that of CBFV. Considering that CFC idea can be extended to study: phase-to-phase CFC, phase-to-amplitude CFC and amplitude-to-amplitude CFC, therefore other two forms of CFC not studied in this work can be further investigated and they both can use different frequency contents from two signals.

Since Brazilian physiologist Leao described the cortical spreading depolarization (CSD) in 1944, there has been significant progress in this area (50). The physiological haemodynamic response to CSD is the dilation of resistance vessels, in order to increase regional CBF to match the energy consumption during the neuronal depolarization phase (51, 52). However, with the dysfunction of local microvasculature, sever microvascular spasm instead of vasodilation is coupled to the neuronal depolarization phase, inducing cortical spreading ischemia, and/or inverse neurovascular coupling. Researchers already demonstrated cortical spreading ischemia in animal models, and in patients with stroke, aneurysmal subarachnoid hemorrhage (51–53), using electrocorticogram (ECoG). Cortical spreading ischemia probably represents a late consequence of prolonged impaired neurovascular coupling. Our current work can be considered as a preliminary and technology development toward providing capability for the first time to continuously monitor and detect impairment in neurovascular coupling—so that spreading ischemia can be predicted. However, this hypothesis remains speculative at the moment and will motivate the field to collaborate and share multimodality recordings to facilitate further development of the algorithms and recording technologies.

We also looked into the changes of CFC between CBV and EEG over time. We reviewed the results of the 16 stroke patients, and in most cases the CFC in gamma and beta bands were continuously higher than other bands, as shown in the Figures S1A–C; however, 5 out of 16 patients did not show continuously strong CFC in gamma and beta bands as shown in Figures S1D–F. Interestingly, two out these five patients died and this bears the speculation that consistently high CFC might be associated with good outcome. However, given the small number of patients studied in the present work, it is not feasible to provide robust statistical conclusion on this topic. More sophisticated study on the changes of CFC over time should be done in the future.

Finally, we acknowledge the following limitations of this study. First, the current study has limited EEG channels and limited spatial discrimination of TCD measured CBFV. Only six EEG channels were used in this cohort of patients. Although, the six EEG channels covered the frontal, occipital and parietal lobes, they were not enough to cover the whole brain hemisphere with adequate, spatial resolution. More channels need to be used to more precisely determine the brain region that shows strongest neurovascular coupling between hemodynamic signals and EEG. Secondly, a small cohort of ischemic stroke patients (only 16 patients) were recruited in this paper, and we did not get adequate ranges of outcome to robustly study the relationship between neurovascular coupling characteristics and patient outcome. A prospective study on a bigger cohort is needed to further establish the clinical values of the EEG-CBFV multimodality monitoring. Furthermore, the study lacks a metabolic assessment to define neurovascular coupling and uncoupling.

## Conclusion

In this study, we found a strong coupling relationship between phase of CBFV slow waves and EEG amplitude in β and γ bands. We also found that the occipital region shows the strongest phase-amplitude coupling between CBFV and EEG. These findings are consistent with other studies. Moreover, we demonstrated the degree of stenosis of stroke patients correlated with the asymmetry of the neurovascular coupling strength between the two brain hemispheres. The PAC metric has the potential for routine use to guide clinical management in patients with occlusive stroke, but further studies are needed.

## Author Contributions

The concept and study design were formed by XH, LL, and XL. Data acquisition was conducted by YP and ZZ. Data analysis was conducted by XL and DW. Drafting of the manuscript and figures was contributed by XL, XH, DW, LL, ZZ, and YP.

### Conflict of Interest Statement

The authors declare that the research was conducted in the absence of any commercial or financial relationships that could be construed as a potential conflict of interest.
