# Supplementary material for: Cross-Frequency Coupling Between Cerebral Blood Flow Velocity and EEG in Ischemic Stroke Patients With Large Vessel Occlusion
Source: Front Neurol. 2019 Mar 12;10:194. doi: 10.3389/fneur.2019.00194 (PMC6422917; doi:10.3389/fneur.2019.00194)
Supplement: Supplementary file 1 [file Image_1.pdf]

Supplementary Figure 1

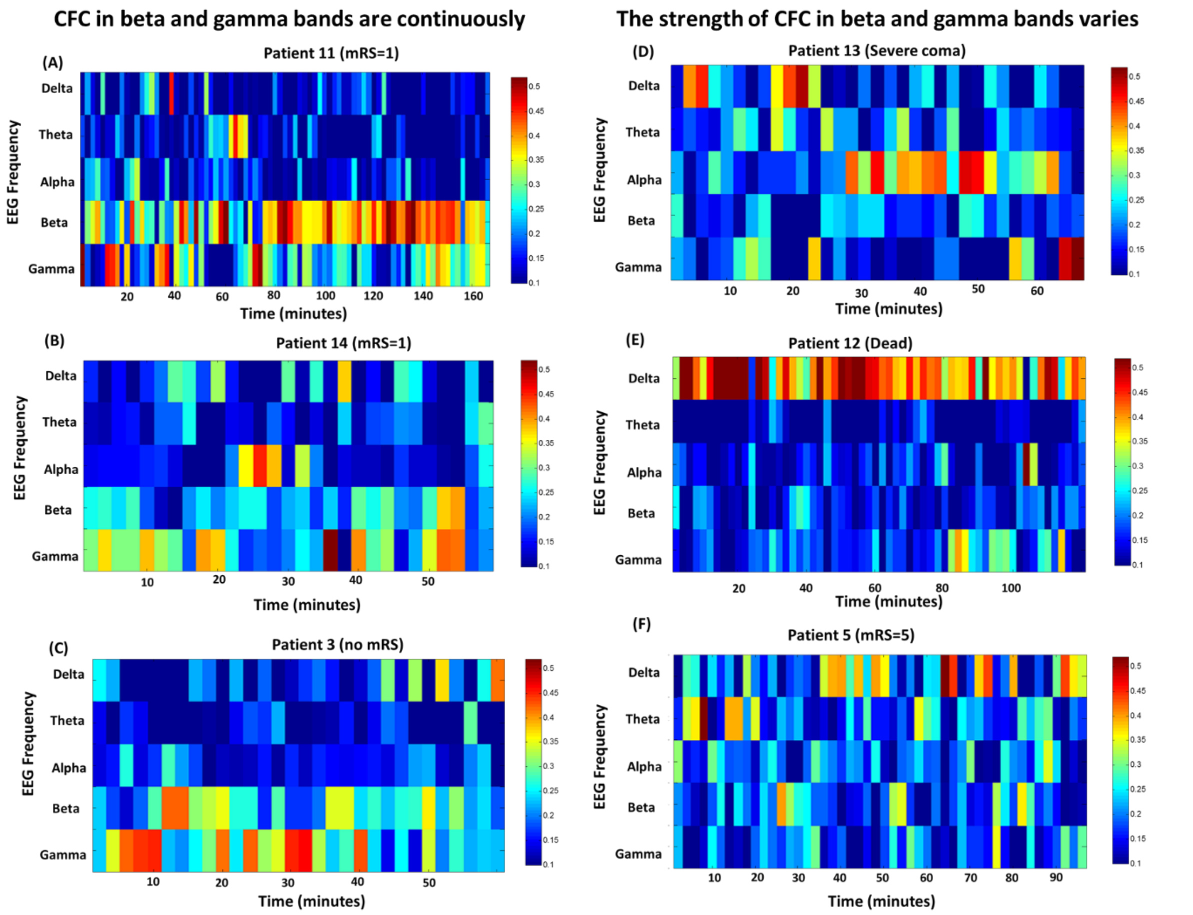

SFig.1 The changes of cross frequency coupling (CFC) between cerebral blood flow velocity and EEG (in five frequency bands) over time for different patients. X axis: time (minute); Y axis: EEG in five frequency bands (δ, θ, α, β, γ). The CFC was continuously higher in beta and gamma bands in patient 11 (A), 14 (B) and 3(C). The strength of CFC varies across frequencies over time in patient 13 (D), 12(E) and 5(F).
